# Effect of Glyceryl Monoolein Addition on the Foaming Properties and Stability of Whipped Oleogels

**DOI:** 10.3390/gels8110705

**Published:** 2022-10-31

**Authors:** Eleftherios G. Andriotis, Paraskevi-Kyriaki Monou, George Komis, Nikolaos Bouropoulos, Christos Ritzoulis, Georgios Delis, Evangelos Kiosis, Georgios Arsenos, Dimitrios G. Fatouros

**Affiliations:** 1 Laboratory of Pharmaceutical Technology, Department of Pharmacy, Faculty of Health Sciences, Aristotle University of Thessaloniki, GR 54124 Thessaloniki, Greece; 2 School of Biology, Aristotle University of Thessaloniki, GR 54124 Thessaloniki, Greece; 3 Department of Materials Science, University of Patras, GR 26504 Patras, Greece; 4 Foundation for Research and Technology Hellas, Institute of Chemical Engineering and High Temperature Chemical Processes, GR 26504 Patras, Greece; 5 Department of Food Science and Technology, International Hellenic University, Alexander Campus, GR 57400 Thessaloniki, Greece; 6 Laboratory of Pharmacology, School of Veterinary Medicine, Faculty of Health Sciences, Aristotle University of Thessaloniki, GR 54124 Thessaloniki, Greece; 7 Clinic of Farm Animals, School of Veterinary Medicine, Faculty of Health Sciences, Aristotle University of Thessaloniki, GR 54124 Thessaloniki, Greece; 8 Laboratory of Animal Husbandry, School of Veterinary Medicine, Faculty of Health Sciences, Aristotle University of Thessaloniki, GR 54124 Thessaloniki, Greece

**Keywords:** oleogels, oleofoams, MCT oil, glyceryl monostearate, glyceryl monoolein

## Abstract

Medium Chain Triglyceride (MCT) oil was successfully combined with Glyceryl Monostearate (GMS) and Glyceryl Monoolein (GMO) to form oleogels that were subsequently whipped to form stable oleofoams. The co-crystallization of GMS and GMO at a ratio of 20:1, 20:2.5, and 20:5 within MCT oil was studied through Differential Scanning Calorimetry (DSC), X-ray Diffraction analysis (XRD), rheological analysis, Fluorescence Recovery after Photobleaching (FRAP), Fourier Transform Infrared Spectroscopy (FTIR), and polarized microscopy. The addition of 5% GMO resulted in the production of more stable oleogels in terms of crystal structure and higher peak melting point, rendering this formulation suitable for pharmaceutical applications that are intended to be used internally and those that require stability at temperatures close to 40 °C. All formulations were whipped to form oleofoams that were evaluated for their storage stability for prolonged period at different temperatures. The results show that oleofoams containing 5% MGO retained their foam characteristics even after 3 months of storage under different temperature conditions.

## 1. Introduction

Foams are colloidal systems containing air in the form of bubbles entrapped in a continuous phase [1]. An in-depth understanding of these systems is of paramount importance. In the cosmetics, food, and pharmaceutical industry, aqueous foams have been extensively used and studied. Foams are considered as metastable systems that are stabilized by low-molecular weight molecules such as surfactants, amphiphilic polymers, proteins, or, alternatively, by dispersed particles [1,2,3]. Oil-based foams (gas-in-oil systems) consisting of edible raw materials have gained a lot of attention due to their obvious potential in the oil structuring field, mainly as an alternative way of producing healthier food products [1]. The stabilizing compound used in such systems should be insoluble to the continuous phase, ensuring in this way the foamability of the system [1,4]. The process for preparing such foams typically involves heating so as to melt a mixture that contains the continuous oil phase (vegetable oil) along with the stabilizing agents (fatty acids, acylglycerols, etc.), until the latter is fully incorporated therein. Subsequently, the system is cooled down following a precise time–temperature protocol, allowing the stabilizing agent to crystallize. The foam is then formed by applying a whipping process that promotes air entrapment within the continuous phase [5]. Whipping process has been reported to be necessary so as to obtain stable foams, since through this process the formed bubbles are covered by a layer of crystals (Pickering monoglyceride crystals) [1,5,6,7,8,9,10,11,12]. Two important factors that strongly influence the final properties of crystal–oil mixtures, especially in the case of monoglycerides, are the duration and the temperature of the process [5].

The aim of the present study is to conduct an in-depth investigation of stable food-grade oleofoams as a viable, relatively fast, and scalable process which, in turn, could be applied to pharmaceutical products for humans or for veterinary applications. These applications include but are not limited to materials that can be used as internal teat sealants (ITSs) in dairy animals. Udder health is a key issue dictating the productivity and longevity of dairy animals as well as the financial viability of farms. In practice, prevention of clinical and sub-clinical intramammary infections (IMI) is a priority that focuses on pathogens entering through the teat canal. The latter is achieved using internal teat sealants (ITSs) at the commencement of the drying-off period. Here, we propose the use of designated stable food-grade oleofoams as components of ITS products for dairy animals [13,14,15]. Although the application of ITSs during the dry period is thoroughly studied, there is a growing demand for new environmentally friendly products that consider “green”, “natural”, and “sustainable” alternatives to existing ITS products. The notion is that these alternative products should have equal levels of IMI prevention without having components such as heavy metals and petroleum-based materials. An interesting resource of materials that could cater to the needs of ITSs are structured vegetable oils (SVOs). The latter include all types of oleogels and oleofoams [16]. The application of SVOs as ITSs is described in a patent (PCT International Application No. PCT/GR2020/000024 filed on 15 May 2020 and published under No. WO2020/229851 on 19 November 2020; European Patent Application No. EP20742414.4 published under No. ΕΡ 3968954 on 23 March 2022) where vegetable oils are structured towards the formation of stable oleogels. These oleogels are subsequently homogenized to form thick pastes that are suitable for intramammary infusion as part of ITSs. The raw materials used in the current study were of natural origin, edible, and of pharmaceutical grade. Glyceryl Monostearate (GMS), Glyceryl Monooleate (GMO), and Medium Chain Triglyceride oil were chosen as raw materials because they are of plant origin, can be obtained at food- and pharmaceutical-grade, are relatively inexpensive, and have been studied extensively. Additionally, Medium Chain Triglycerides are reported to have antimicrobial properties [17], thus making them a very promising material for the creation of products with antimicrobial properties. These materials were combined to produce oleogels by using different cooling rates, with a focus on the incorporation of GMO. The oleofoams were prepared in two steps, namely the preparation of stable oleogels and their subsequent aeration by whipping to produce stable oleofoams. The study of both oleogels and oleofoams is considered very important, as the physical and structural properties of the initial oleogels play a significant role in the final properties and stability of the produced oleofoams. Thus, in this study both oleogels and oleofoams comprised of GMS, GMO, and MCT oil mixtures were thoroughly studied. The effect of the cooling rate on the oleogel microstructure was investigated through rheometry, Differential Scanning Calorimetry (DSC), Polarized Light Microscopy (PLM), Confocal Laser Scanning Microscopy (CLSM), Fourier Transform Infrared Spectroscopy, and X-ray Diffractometry (XRD). The optimum conditions were determined for the preparation of oleofoams, with a focus on foamability and foam stability.

## 2. Results and Discussion

### 2.1. Thermal Behavior during Cooling

The thermal behavior of the different oleogel formulations during cooling was assessed using Differential Scanning Calorimetry (DSC). The application of different cooling rates was used as a simulation of the cooling process during oleogel preparation. The cooling profile applied for oleogel preparation is illustrated in Figure S1 (in Supplementary Information, SI). According to the first derivative plot, the cooling rate is ≤1 °C/min for temperatures ≤ 60 °C; it could thus be simulated by experiments conducted at this cooling rate. Higher cooling rates (up to 20 °C/min) were also applied to simulate quenching conditions (rapid cooling). The interpretation of thermograms was based on the respective DSC thermograms of the raw materials (Figures S2 and S3).

**Figure 1:**
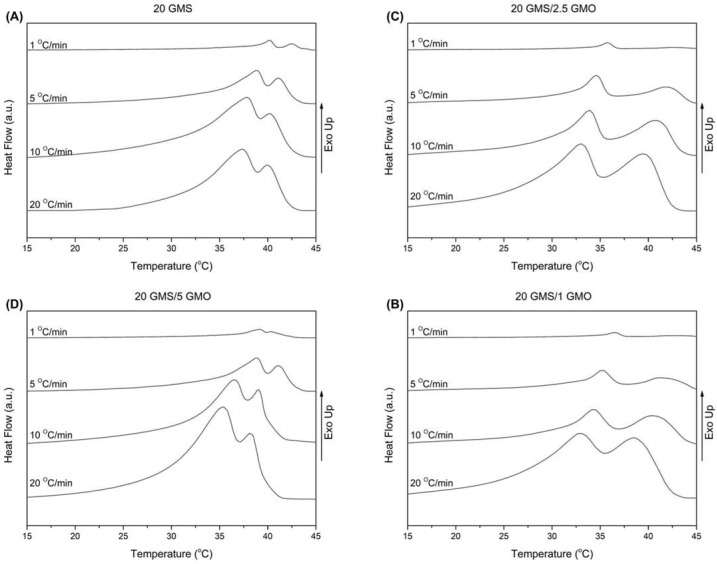
DSC cooling curves for cooling rates of 1, 5, 10, and 20 °C/min for (**A**) 20 GMS, (**B**) 20 GMS/1 GMO, (**C**) 20 GMS/2.5 GMO, and (**D**) 20 GMS/5 GMO.

The thermal behavior of the different oleogel formulations during cooling at non-isothermal conditions is presented in Figure 1A–D. The samples were heated to 80 °C and then cooled to 15 °C at varying cooling rates (1, 5, 10, and 20 °C/min). All thermograms were characterized by the presence of two wide peaks, indicative of the transition from the crystalline phase towards the stable sub-α phase [18].

In Table S1, the peak crystallization temperatures for the two observed peaks (denoted as peak I and peak II) are tabulated along with the total crystallization enthalpies. According to Figure S5, peak II appears at a higher temperature during cooling and is attributed to the formation of the inverse lamellar phase [18]. The 1% and 2.5% GMO-containing samples exhibited close peak temperatures for all cooling rates, while peak II of the 20 GMS/5 GMO sample appears at a lower temperature for the 1 °C/min cooling rate. It is safe to assume that, in a predominantly triglyceride liquid, monoglycerides such as GMO and GMS preferentially adsorb at the air–fat interface, where the outward orientation of their polar moieties is not detrimental to the bulk hydrophobic structure. This is such a strong tendency that GMO and GMS can displace proteins from the oil–water interface in ice cream models [19]. Thus, any enthalpic changes brought by the addition of the monoglycerides are due to the latter’s restructuring close to the air–fat surface. Under this light, cooling rate changes could be attributed to the higher interfacial concentration of GMO, leading to a different surface structuring compared to the other formulations which could involve the formation of two distinct phases at the air–fat interface, one created by GMS and the other by GMO. On the other hand, peak I appears at a lower temperature compared to peak II, indicating the formation of the sub-α crystalline phase [1,18]. In this case, the samples appear to be divided into two groups with similar behavior (20 GMS with 20 GMS/5 GMO and 20 GMS/1 with 20 GMS/2.5 GMO). In the case of 20 GMS/1 GMO and 20 GMS/2.5 GMO samples, the observed peak temperature for peak I is lower compared to the respective peak temperature of peak II for all the applied cooling rates. On the other hand, both 20 GMS and 20 GMS/5 GMO samples exhibit similar values for peak crystallization temperatures (peak I and peak II) for the 1 °C/min cooling rate, indicating that the transition to the sub-α crystalline phase is faster. The addition of GMO seems to increase the value of Δ*Hc* in the case of the 20 °C/min cooling rate, indicating differences in packing densities when there are two crystal populations compared to the single crystal population in the case of 20 GMS. These packing differences should be attributed to the inability of the two hydroxyls of each glycerol of GMO and GMS to become involved in hydrophobic interactions with MCT; at lower cooling rates, GMS and GMO would have the time to diffuse and adsorb at the interface.

To obtain further insights on the crystallization kinetics of the formulations, DSC data were converted into relative crystallinity and plotted against the temperature using the following equation [20]:

(1) $$X\left(T\right)=\frac{\int_{T_{0}}^{T}\left(\frac{dH_{c}}{dT}\right)dt}{\Delta H_{c}}$$

where 𝑇_0_ and 𝑇 are the onset and an arbitrary temperature, respectively, 𝑑𝐻_𝑐_ is the crystallization enthalpy released during an infinitesimal temperature interval 𝑑𝑇, and Δ𝐻_𝑐_ is the total enthalpy of crystallization at a certain cooling rate. Temperature scale can be further converted to time using the following equation [20]:

(2) $$t=\frac{\left|T_{0}-T\right|}{\varphi }$$

where *T* is the temperature at time *t* and φ is the respective cooling rate in °C/min.

**Figure 2:**
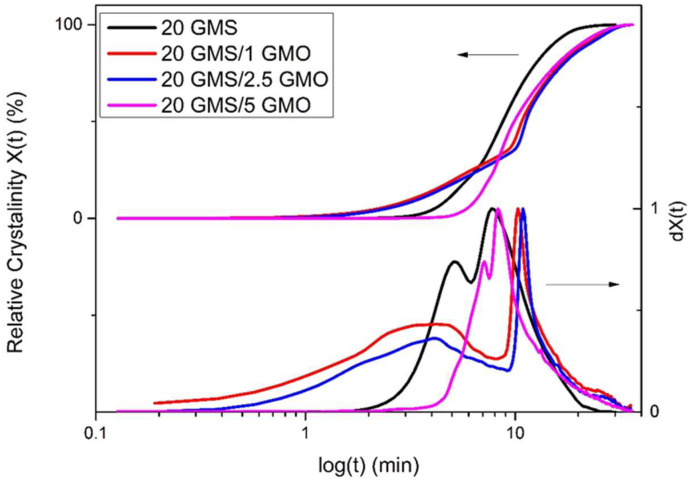
Relative crystallinity (X(t)) and first derivative of relative crystallinity (dX(t)) (quantified by DSC data) vs. time at cooling rate of 1 °C/min.

The relative crystallinity vs. temperature plots (Figure S4A,C,E,F) exhibit the typical inverse sigmoidal shape, which indicates a rapid early-stage crystallization followed by a slower later stage crystallization. The increase in the cooling rate is followed by a broadening of the overall process temperature range. Additionally, the relative crystallinity vs. time plots (Figure 2 and Figure S4B,D,F,G) offer insights regarding the crystallization process, as the different crystallization steps are more distinct. Specifically, curves obtained for cooling rates higher than 1 °C/min have a typical sigmoidal shape, with a steep slope and no lag time. On the contrary, the curves obtained for 1 °C/min exhibit different characteristics for the different samples, with 20 GMS/1 GMO and 20 GMS/2.5 GMO having similar profiles.

To interpret these observations, a working hypothesis is suggested based on the previous discussion: GMO (in small amounts) is introduced in the form of individual molecules within the inverse lamellar phase formed by GMS molecules, creating structurally defective sites due to GMO’s hydrophobic tail-different conformation (always compared to GMS). The initiation of rapid crystallization could be attributed to these individual molecules, acting also as nucleation sites. The presence of the suggested structural defects could plausibly explain the fact that the peak melting points of 20 GMS/1 GMO and 20 GMS/2.5 are not affected during storage, suggesting the inhibition of β crystal formation. On the other hand, 5% *w/w* of GMO appears to be a sufficient concentration for GMO to be organized in separate interfacial domains (see the previous discussion), phase-separated from those of GMS. This could explain the similar DSC cooling profiles of 20 GMS and 20 GMS/5 GMO samples; it is backed by the adsorption kinetics at high cooling rates and existing experience with similar systems [21,22]. The closest equivalent is the orogenic displacement of proteins by small-molecule emulsifiers at the interface, where a cohesive surface layer is broken down by the partial displacement of proteins by small-molecule emulsifiers, which is then followed by the creation of a 2D fluid interface (of the extensive relevant literature, see [23] for emulsions and [24] for foams). To the best of the present authors’ understanding, this is the first report of an orogenic-type displacement in oleofoams.

Half-time crystallization (*t*_1/2_) as a function of cooling rate φ (Figure S5) is defined as the time needed from the onset of the crystallization process to the time at which the relative degree of crystallinity reaches 50% [25]. This value is lower for 20 GMS and 20 GMS/5 GMO samples for lower cooling rates; thus, these two samples have higher crystallization rates. The fact that 20 GMS has the lowest t_1/2_ value for all given cooling rates means that the addition of GMO decelerates the crystallization of GMS, but not in a concentration-dependent way. This is consistent with the orogenic-type disruption of GMS by limited amounts of GMO. The different t_1/2_ values of the different formulations could also be related to different crystallization processes, as has previously been hypothesized.

### 2.2. Rheological Studies

The rheological behavior of the formulations (in oleogel form) during cooling at different cooling rates was also studied. Temperature sweep tests were performed according to the literature [26] in order to monitor the evolution of complex viscosity during the cooling process and subsequently convert these data to time-relevant plots based on the following expression of relative crystallinity (quantified by rheology) [26,27]:

(3) $$\frac{Y_{s}}{Y_{\max}}=\frac{\eta^{*}\left(t\right)-\eta_{\mathrm{Oil}}}{\eta_{\max}^{*}-\eta_{\mathrm{Oil}}}$$

where η^*^(t), η^*^_max_, and η_oil_ are the complex viscosity at time t, the maximum complex viscosity, and the complex viscosity of the oil used (MCT Oil), respectively.

Figure S6A,C–E show similar patterns for all samples for the same cooling rate, which only differ by the cooling rate that was applied. This again suggests that all the observed enthalpic events relate to interfacial, not bulk, phenomena (the reader is reminded that interfacial phenomena play, in general, a limited role in bulk colloidal shear rheology). The decrease in the cooling rate is followed by a shifting of the curves towards higher temperatures. This shifting is expected as there is more time available for the system to form and evolve crystal structures (crystal growth step) [20]. These data were expressed as relative crystallinity and plotted against time, revealing similar crystallization profiles. All sample curves had a typical sigmoidal shape with no significant alterations in their slopes, while a distinct lag time is present for the curves obtained with the 1 °C/min cooling rate. Since it is not related to interfacial processes, this lag time is of different physical meaning to the one discussed in DSC analysis, as it represents a period where formed crystals have not yet grown to the extent that they could influence the measured complex viscosity of the system.

### 2.3. Effect of Cooling Rate on Crystal Formation

**Figure 3:**
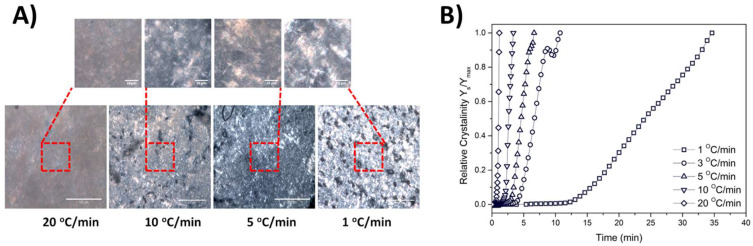
Typical example of relative crystallinity (quantified by rheology data) plotted as a function of time for the 20 GMS/5 GMO sample at cooling rates from 1 to 20 °C/min (**A**), alongside the respective polarized micrograph obtained at the end of each measurement, at two different magnifications (red square area is magnified at the respective micrograph) (**B**).

All samples were observed by polarized optical microscopy upon the completion of the cooling process. Specifically, following the temperature sweep test, the samples were directly transferred from the rheometer apparatus and gently placed on a microscope glass slide. The samples were covered with a thin cover slip and the observation performed at 25 °C. All samples were covered by a thin cover slip after a period of 30 min to ensure that they had reached ambient temperature. Figure S7 shows the crystal texture of the samples obtained for different cooling rates at two magnification levels. The micrographs depicted in Figure S7A show that the crystals (elongated plate domains) that were formed had a more elongated shape as the cooling rate decreased, as was expected [26]. The crystal formations for the 20 GMS samples at higher cooling rates (10–20 °C/min) are not clearly visible compared to lower cooling rates (1–5 °C/min), indicating that the crystal formation process is not fully completed during the experiment at conditions that simulate quenching (rapid cooling). The addition of 1% and 2.5% *w/w* of GMO seem to further inhibit the formation of larger crystals. The phenomenon is reversed in the case of the 20 GMS/5 GMO sample, where the crystal formations are also visible at a 10 °C/min cooling rate. This observation is in close agreement with the DSC findings related to the 20 GMS/5 GMO sample and could also be related to the mechanism that was previously hypothesized (time-dependent limitations of GMO and GMS diffusion/adsorption). Figure S7B shows the same samples at a lower magnification level, which allows for observation of the solid crystal clusters dispersed in a continuous oil phase that are attributed to slow re-crystallization and phase separation from the residual triglycerides [18]. In a way similar to Figure S7A, the crystal clustering for 20 GMS/5 GMO starts at higher cooling rates (10 °C/min) compared to the 20 GMS sample, while the addition of 1% and 1.5% *w/w* GMO decelerates the phenomenon. This observation is indicative of the formation of more stable crystalline structures following the addition of 5% GMO. Figure 3 shows a typical example of the study of relative crystallinity vs. time as it is derived from the rheological studies for the oleogel sample of 20 GMS/5 GMO. The relative crystallinity for different cooling rates was followed by inspection of the crystalline structures via polarized light microscopy, as presented in Figure 3 which shows the 20 GMS/5 GMO oleogel sample at two different magnification levels.

### 2.4. Effect of Storage Conditions

The previously discussed findings emphasize not only the strong dependency of the crystal formation process on the cooling rate, but also the importance of the final cooling temperature and the time that the system stays at this temperature [1]. To evaluate the effect of the storage conditions on the formulations under study, the prepared samples were tested for their melting behavior by means of DSC analysis. Specifically, the formulations were prepared by heating at 80 °C and cooling to 4 °C at a 1 °C/min cooling rate. All formulations were tested after being stored at 4 °C for 24 h.

**Figure 4:**
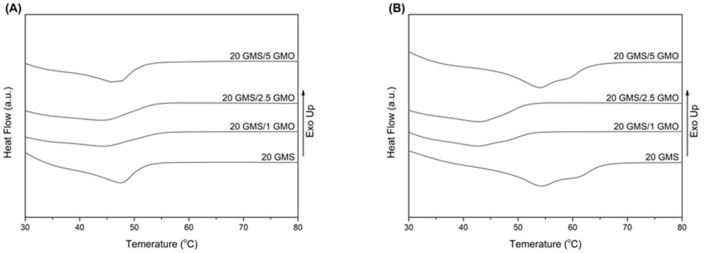
DSC thermograms of heating curves for the different formulations: (**A**) 0 h stored at 4 °C and (**B**) 24 h stored at 4 °C (heating rate 10 °C/min).

Figure 4A shows the melting curves of the formulations directly after they were cooled to 4 °C at a cooling rate of 1 °C/min (freshly prepared), while Figure 4B shows the melting curves of the formulations after being stored at 4 °C for 24 h. The peak melting point values are summarized in Table S2. The freshly prepared samples exhibit a single broad transition peak, with 20 GMS and 20 GMS/5 GMO samples having higher peak melting temperatures (Figure 4A). The absence of two distinct peaks corresponding to the melting of the inverse lamellar phase at higher temperatures and the melting of the sub-α crystals at lower temperatures [18] are indicative of the presence of overlapped peaks, possibly meaning that the system is transitioning to the more stable sub-α phase during the cooling process [18]. The lower overall peak melting temperature, compared to pure GMS (60.05 °C), is attributed to MCT oil acting as a solvent and plasticizing the system [28].

Figure 4B shows the melting peaks of the samples that were stored at 4 °C for a period of 24 h. The obtained thermograms reveal an increase in the peak melting point for 20 GMS and 20 GMS/5 GMO samples (Table S2), while the peak melting point of 20 GMS/1 GMO and 20 GMS/2.5 GMO samples appears unaffected. This increase is typical of the transition of the sub-α crystals to the more stable β crystals that melt at higher temperature [18]. The distinct peak shoulder for both formulations is indicative of the presence of two different crystal phases, meaning that the transition towards β crystals is not fully completed [18]. The fact that the melting point of 20 GMS/1 GMO and 20 GMS/2.5 GMO samples is not affected is probably an indication of the different crystallization rates of the monoglycerides (caused by the presence of smaller amounts of GMO) and of the localization of phase-compatible GMO–GMS at the interface, as was previously hypothesized.

**Figure 5:**
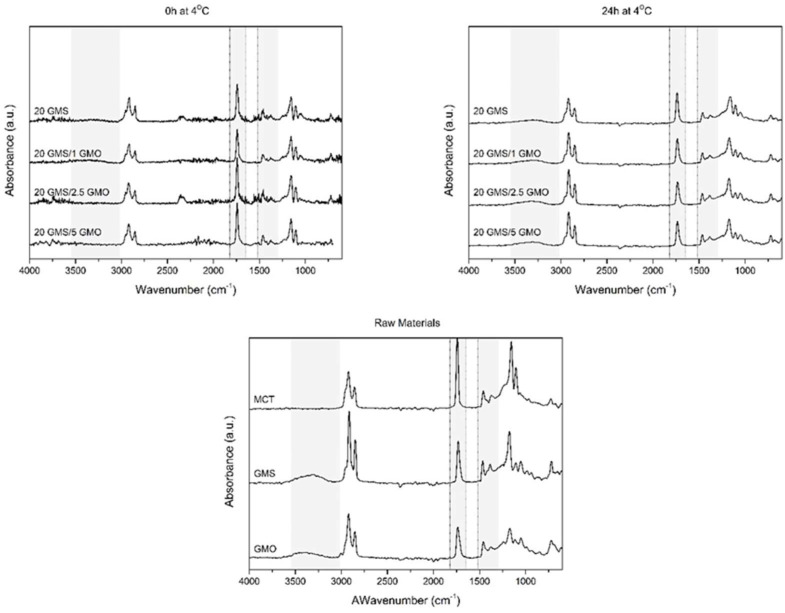
FTIR spectra of oleogels after cooling to room temperature (t = 0 h at 4 °C) and after 24 h of storage at 4 °C (t = 0 h at 4 °C) alongside the spectra of raw materials.

The effect of storage conditions was also evaluated by ATR-FTIR spectroscopy. Samples were studied directly after cooling at 4 °C and after 24 h of storage (Figure 5). The light gray highlighted areas are representative of the regions of interest. The high energy region between 4000 and 3000 cm^−1^ is assigned to the hydrogen bonds formed by the OH groups of the monoglycerides [18,28]. The free hydroxyl groups of monoglycerides, corresponding to position 2 and 3 of their monoacylglycerol backbone, are involved in the formation of intra- or inter-molecular hydrogen bonds. The presence of a wide peak around 3600–3100 cm^−1^ for the samples that were stored for 24 h at 4 °C reveals the coexistence of inter- and/or intra-molecular hydrogen bonding [18,28]. The fact that only the oleogel samples that were stored at 4 °C for 24 h exhibited these peaks is an indication that these types of bonds require time to form properly; thus, storage time positively affects the properties of the produced oleogels, rendering them more stable and capable of withstanding the foaming process. Additionally, the presence of smaller peaks for (hydroxyl-free) MCT should be attributed to the presence of limited amounts of water and limited triglyceride hydrolysis. The formation of these hydrogen bonds is essential for the structuring of the system and for the creation of a coherent interfacial network.

Peaks found around 1735 cm^−1^ are attributed to C=O stretching vibrations of carbonyl groups belonging to the ester bonds between glycerol and fatty acids [28]. All spectra show a single sharp peak at this region which is not affected by the addition of GMO or the different storage conditions. This peak is characteristic of the presence of MCT oil, which is the dominant material (highest concentration) in all formulations. Peaks corresponding to C-H bending vibrations are present in the region between 1400 and 900 cm^−1^ for all spectra [28]. These peaks are also not affected by the addition of GMO or the different storage conditions, as they are associated with hydrophilic dispersion forces which are omnipresent in these systems (in fact they are expected to be the dominant contributors to the structural integrity of oleogels).

### 2.5. Stability against Phase Separation

To monitor stability and evaluate the stability of the formulations against possible face separation phenomena, the oleofoams and oleogels under study were analyzed by Fluorescence Recovery After Photobleaching (FRAP). Samples prepared by the 1 °C/min cooling rate and 24 h of storage at 4 °C were tested before and after foaming by whipping. Changes in the recovery rate along with the fraction of immobile molecules could provide useful insights on the nature of the systems under study. The quantification of these values was performed by fitting an exponential model to the normalized FRAP data [29]:

(4) $$y\left(t\right)=A\left(1-e^{-\tau t}\right)$$

where A, *τ*, and t correspond to the maximum intensity (plateau intensity), a fitted parameter, and recovery time after bleach, respectively. The time required to reach 50% of the full recovery is referred as t_1/2_ and it is indicative of the recovery speed. This value is quantified by applying the following Equation [29]:

(5) $$t_{1/2}=\frac{ln\left(0.5\right)}{-\tau }$$

The mobile fraction can be quantified by the value of plateau intensity (A), thus the immobile fraction (*IF*) can be expressed as [29]:

(6) IF(%)=100×(1−A)

**Figure 6:**
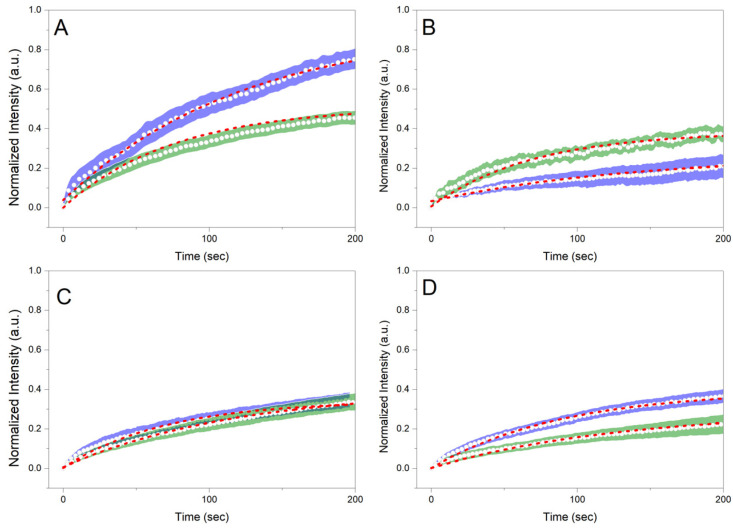
Normalized recoveries (FRAP) plotted against recovery time for (**A**) 20 GMS, (**B**) 20 GMS/1 GMO, (**C**) 20 GMS/2.5 GMO, and (**D**) 20 GMS/5 GMO samples. Green and blue highlighted curves represent samples before and after the whipping process, respectively. Red highlighted curves represent the fitted exponential model.

**Figure 7:**
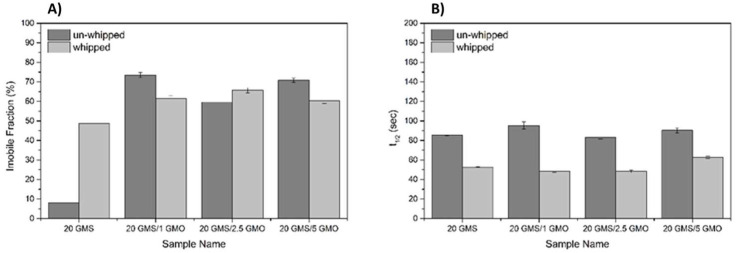
Immobile fraction (IF) (**A**), and *t*_1/2_ values (**B**) for the different formulations as quantified by FRAP analysis.

Figure 6 shows the normalized recoveries plotted against the recovery time for the different samples before and after whipping/foaming. For 20 GMS samples, the whipping process results in a significantly higher immobile fraction value (Figure 7A) compared to the homogeneous un-whipped sample. This observation can be a strong indication of GMS adsorption at the interface, where its hydroxyls are no more detrimental to (dispersion force-dominated) bulk structural integrity. This phase separation is representative of the inferior mechanical properties of this type of material. The presence of phase separation is also hinted by the higher *t_1/2_* value of 20 GMS un-whipped samples (Figure 7B). The addition of even small amounts of GMO inhibits mobility, hinting at its role as a crystallization nucleator. The selective adsorption of GMO and GMS at the interface does not appear to affect such processes. In all cases, introduction of GMO and/or GMS results in the reduction of *t*_1/2_, which is in line with the mobility decrease for molecules that are confined to an interface.

### 2.6. Effect on Crystal Structures

**Figure 8:**
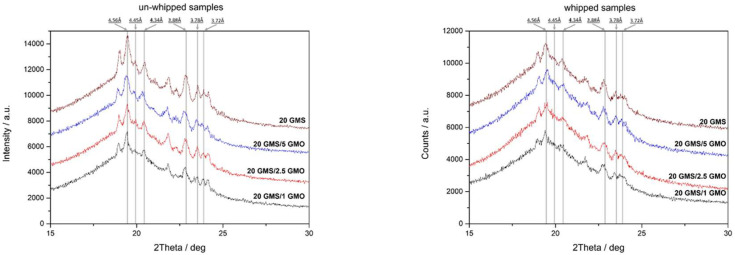
XRD patterns of whipped and un-whipped samples.

Whipped and un-whipped samples were studied by XRD analysis to determine and evaluate the crystalline phases present in the system (Figure 8). Both types of samples have similar patterns indicating that the foaming process does not alter the crystalline structure of the samples. Additionally, both types of samples exhibited the typical XRD pattern of the short spacings of orthorhombic sub-α packing (0.417 nm, multiple from 0.408 nm to 0.364 nm) [1]. Sub-α and α crystals are not in a thermodynamically stable state and are expected to undergo polymorphic transition to β crystals. The sub-α crystalline phase can either transition to an α-alpha polymorph, and subsequently to the most stable β-polymorph, or transition directly to latter [1]. The diffractographs in Figure 8 reveal the presence of both sub-α and β crystals (0.360, 0.370–0.390, and 0.460 nm) [1]. The presence of multiple crystalline phases indicates that the transition to β crystals is an on-going process that has not been fully completed at the time of the experiment. Additionally, the presence of multiple crystalline phases including α crystals could possibly explain the lower peak melting temperature (DSC heating curves) of 20 GMS/1 GMO and 20 GMS/2.5 GMO after 24 h of storage at 4 °C, as was previously discussed. Of interest is the loss of crystallinity after foaming. This is in line with the inability of interfacially adsorbed GMO and GMS to exert a 3D crystal network, and further supports the original hypothesis that these molecules selectively reside at the air–fat interface.

### 2.7. Storage Stability Assessment

The storage stability of the different foam formulations under different storage conditions was also evaluated. The foam samples were stored for 3 months at 4, 25, and 39 °C, simulating three different working temperatures representative of cooling storage chambers, shelf storage, and body temperature (human or animal), respectively. Stability was qualitatively evaluated by polarized microscopy that could provide useful information concerning the state of the entrapped air and possible bulk phase separation. Figure S11 shows the type of air bubbles that were formed during the whipping process along with qualitative information concerning the air distribution within the bulk of the material. Based on these micrographs, all samples exhibit air bubbles of relatively small size, with the 20 GMS/5 GMO sample having smaller bubbles. As discussed in the previous sections, at this GMO concentration, a coherent 2D phase-separated interface is formed, which can exert adequate stabilization. The time-dependent evolution of the shape and distribution of the air bubbles show the action of permanent destabilization mechanisms such as Ostwald ripening (“disproportionation”) and/or coalescence phenomena. All samples have a very distinct crystalline structure, with crystal formations present on the exterior and interior area of the/bubbles (except for the 20 GMS/2.5 GMO sample where the crystalline structure is not that clear) while regions of low light intensities attributed to phase separation (free oil regions) are also present. This is in line with the phase incompatibility between GMO and GMS; as discussed earlier, the two phases become compatible through an orogenic-type mechanism at 5% GMO.

**Figure 9:**
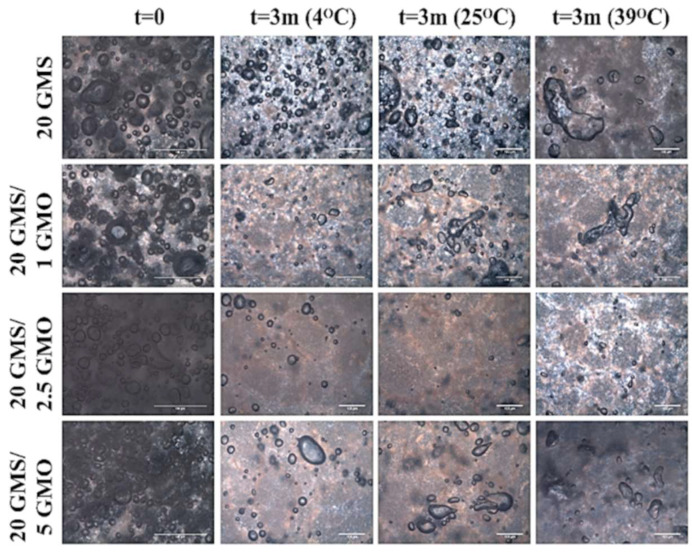
Polarized Light Microscopy micrographs of foams stored for three months under different storage conditions.

Figure 9 summarizes the micrographs obtained for the different foam formulations after a period of three months and under different storage conditions. The air bubbles are expected to ripen/coalesce, shrink, and, finally, disappear, as in the case of the 20 GMS/2.5 GMO sample. More specifically, the 20 GMS/2.5 GMO sample partially retained its initial foam structure when stored at 4 °C, but the amount of entrapped air was minimal. On the other hand, the foam structure was no longer present at the other two storage temperatures, where the air bubbles were absent and signs of intense phase separation were present (dark islets attributed to free oil). The same observations could be made for the 20 GMS/1 GMO sample, except for the presence of some remaining air in the form of coalesced bubbles. On the other hand, the 20 GMS and 20 GMS/5 GMO samples retained their foam structure, even though the air bubbles shrunk significantly and there was intense bubble coalescence/disproportionation. As an overall performance evaluation, the 20 GMS/5 GMO sample retained its foam structure to a greater extent compared to the other formulations. This conclusion is deduced by taking under consideration the completion of the orogenic phase separation at this GMO concentration.

## 3. Conclusions

In this study, the successful preparation of oleofoams composed of food-grade and pharmaceutical-grade raw materials is presented by combining MCT oil with GMS and GMO. The foam preparation process was divided into two steps which included oleogel preparation and subsequent aeration by whipping, leading to the production of oleofoams. The study of the produced oleogels revealed the dependency on the structural characteristics of the foams in terms of the percentage of incorporated GMO, along with the cooling rate and storage conditions (prior to foaming). The addition of GMO had a positive effect on the crystallization rate of the prepared oleogels when added at a concentration of 5% *w/w*. The increase in the peak melting point of the oleogels after 24 h of storage at 4 °C was considered very important as it is indicative of more stable systems. The prepared oleogels were aerated by whipping to produce stable foams with structural integrity that exhibited enhanced thermal stability (elevated melting point) and stability against phase separation. All the above combined with the enhanced storage stability of the oleofoams renders them a very promising product for various applications including pharmaceutical and veterinary applications.

## 4. Materials and Methods

### 4.1. Oil Monoglyceride Mixturs Preparation

As shown in Table 1, predetermined amounts of the tested monoglycerides (Glycerol monostearate 40–55 (Type I, EP), GELEOL, Gattefossé, Saint-Priest, France; Glycerol monooleate (Type 40, EP), PECEOL, Gattefossé, Saint-Priest, France) were added to MCT oil (Medium Chain Triglycerides, EP, LABRAFAC LIPOPHILE WL 1349, Gattefossé, Saint-Priest, France) in a glass beaker and heated to 80 °C under continuous magnetic stirring until a clear homogeneous solution was obtained. The molten mixture was then transferred to 50 mL centrifuge tubes and cooled to room temperature before any further analysis.

### 4.2. Oleofoam Preparation

The oleofoam formulations were prepared according to the literature with some modifications [1,5,28]. Briefly, a predetermined amount of monoglycerides (Table 1) was mixed with MCT oil at 80 °C in a glass beaker under constant magnetic stirring until a clear homogeneous mixture was formed. The mixture was stirred at 80 °C for 30 min to ensure thermal history elimination (complete melting of monoglyceride crystals). The melted mixture was then transferred into the stainless-steel vessel of a bench-top planetary mixer equipped with a balloon-type whisk (AEG KM4000 Ultramix Kitchen Machine, 2.9 lt vessel). Batches (1 Kg) were prepared so as to ensure that 50% of the balloon-type whisk was immersed in the mixture. The bench-top planetary mixer was operating at the lowest speed (scale 1) and the system was cooled to room temperature (25 °C) under constant mixing. The temperature of the formulation was monitored with the immersion thermocouple in the mixture. Upon completion of the cooling process, the formulation took the form of a flowing viscous cream-like froth. The system was kept for 5 h at room temperature (25 °C) without mixing until its complete solidification. The solid formulation was then stored at 4 °C for 24 h. All samples were equilibrated at room temperature for 30 min prior to the foaming process. The whipping process was performed by a bench-top planetary mixer equipped with a balloon-type whisk, set at medium speed (scale 5) for 5 min. A thick foam was formed, which was then transferred to food contact-grade PET containers and stored at 4 °C for further analysis. All containers were sealed with suitable food contact-grade PET lids after they were completely filled with the produced oleofoams in a way that ensured minimum air presence between the lid and the oleofoams. 

**Table 1:** Compositions of different oleogel formulations.

	**Oleogel Composition**		
**Sample Name**	**MCT Oil** **(% *w*/*w*)**	**GMS** **(% *w*/*w*)**	**GMO** **(% *w*/*w*)**
20 GMS	80.0	20.0	-
20 GMS/1 GMO	79.0	20.0	1.0
20 GMS/2.5 GMO	77.5	20.0	2.5
20 GMS/5 GMO	75.0	20.0	5.0

### 4.3. Differential Scanning Calorimetry (DSC) Measurements

The thermal behavior of the prepared formulations (in the form of oleogels) during heating and cooling was assessed by Differential Scanning Calorimetry (DSC) using a Netzsch DSC 204 F1Phoenix heat flux (NETZSCH, Bayern, Germany) according to previously reported studies with some modifications [5,28]. Briefly, a specific amount (5 mg) of sample was placed in DSC aluminum pans and heated to 80 °C at a heating rate of 10 °C/min. An empty DSC aluminum pan was used as the reference sample. The system remained at this temperature for 5 min to eliminate any thermal history. The samples were then cooled down to 10 °C at different cooling rates, whereas raw materials were cooled at a cooling rate of 10 °C/min. Peak melting temperature (*T*_pm_) and apparent melting enthalpy (Δ*H*_m_, J/g) of the different samples were obtained from the endothermic peaks of the thermograms, while thermal behavior during crystallization was assessed from the exothermic peaks of the thermograms. The DSC instrument was operating under a constant nitrogen flow (50 mL/min). Instrument calibration was performed using high-purity benzophenone, indium, and tin. NETZSCH Proteus^®^© Thermal Analysis software package version 5.2.1 (NETZSCH, Bayern, Germany) was used for analysis of the obtained thermograms.

### 4.4. Rheological Studies

Rheological measurements were conducted using an Anton Paar MCR 92 (Anton Paar GmbH, Graz, Austria) strain rate-controlled rheometer equipped with a Peltier module. Parallel-plate geometry (25 mm diameter) with a 0.5 mm gap was selected for all rheological experiments. Oscillatory temperature sweep tests (strain 0.05%, frequency 10 rad/s) were carried out from 80 °C to 4 °C at different cooling rates of 1 to 20 °C/min.

### 4.5. Polarized Light Microscopy Studies

The microstructure of the prepared formulations was studied using an Olympus BX 41 polarized light microscope (Olympus Corporation, Tokyo, Japan). All measurements were performed at an ambient temperature (25 °C). The samples were placed on microscope glass slides and covered by a thin cover slip to form a thin uniform film [1].

### 4.6. Fourier Transform Infrared (FTIR) Spectroscopy Measurements

The reflection FTIR spectra of the prepared oleogels, as well as the raw materials used, were recorded using a Shimadzu IR Prestige-21 spectrometer (Shimadzu, Kyoto, Japan) with a horizontal Golden-Gate MKII single reflection ATR system (Specac, Kent, UK) equipped with ZnSe lenses. Each sample was scanned sixty-four times at 4 cm^−1^ resolution over the wavenumber range of 4000–800 cm^−1^ after appropriate background subtraction. The commercially available IR Solutions (Shimadzu, Japan) software was used to process the spectral data.

### 4.7. X-ray Diffractometry (XRD) Measurements

The crystallinity status of the samples under study was studied by XRD analysis, which was performed by an X-ray diffractometer (Bruker D8-Advance) equipped with a LynxEye detector. Cu Kα radiation (λ = 0.154059 nm), operated at 40 kV and 40 mA, was used. Data were collected over the 2θ range from 5° to 50° at a scanning speed of 0.35 s/step and with a step size of 0.02°.

### 4.8. Fluorescence Recovery after Photobleaching (FRAP) Analysis

FRAP analysis was performed using a Zeiss LSM780 CLSM (Zeiss, Oberkochen, Germany) equipped with a 40×/1.3 NA oil immersion lens. The experimental procedure was performed according to previously reported studies with some modification [29,30]. Samples were prepared by adding 0.01% *w/w* of the lipophilic fluorescence dye (Nile Red, maximum λ*_exc_* = 515 nm and maximum λ*_em_* = 585 nm, Sigma-Aldrich, MO, USA) to the MCT oil phase. The samples (oleogels and oleofoams) were sandwiched between two round coverslips mounted in an Attofluor™ cell chamber (ThermoFisher Scientific, Waltham, MA, USA) forming a uniform thin film with a thickness of ≤0.5 mm. Normalized recoveries of at least 15 Regions of Interest (ROIs) per sample were averaged and plotted against recovery time to monitor the behavior of samples under different experimental conditions. For excitation/bleaching of Nile Red, the 514 nm line of an argon laser was used. Briefly, 10 images were acquired before bleaching to record the initial emission fluorescence intensity. Subsequently, the selected ROIs were repeatedly bleached at 100% laser power and, after bleaching became apparent (at ca. 50% of the initial emission intensity), recovery of fluorescence was monitored until a plateau was reached. For acquisition of the recovery curve, laser power was set to 2% of the maximum output. For both bleaching and recovery, pinhole size was set at 160 Airy units and the scanning frame rate was always between 0.5 and 0.7 frames per second. FRAP curves normalized at values between 0 and 1 were plotted against time and, from such curves, the half-time of recovery (t_1/2_) and the mobile and immobile fractions were deduced. ROI drawing, and the entire experimental setup, was manually set in the FRAP module of Zeiss Zen 2011 software (Black version) (Zeiss, Oberkochen, Germany).

### 4.9. Oil-Binding Capacity (OBC) Studies

Oil-binding capacity was calculated according to the literature [31]. Briefly, 2 mL centrifuge tubes were filled with 1 g of sample and subjected to centrifugation at 4000 rcf (Labofuge 400R, Thermo Fisher Scientific, Waltham, MA, USA) for 15 min and 30 min. After each centrifugation cycle, the superficial drained oil was decanted and the mass of the sample was determined gravimetrically. Oil-binding capacity was determined as the percentage of oil drained after each centrifugation cycle. All measurements were performed in triplicate. The analysis of OBC is reported in the Supplementary Materials.

### 4.10. Overrun Measurements

The overrun measurements are direct indications of the amount of air incorporated in the samples after the aeration/whipping process. A 50 mL plastic vial was filled with the prepared foam and its weight was determined. The same 50 mL plastic vial was subsequently filled by the respective oleogel (in a molten state) and its weight was also determined. The overrun was expressed as [1]:

Overrun (%) = 100 × (m_oleogel_ − m_oleofoam_)/m_oleofoam_ (7)

where m_oleogel_ and m_oleofoam_ are the gravimetrically determined masses of 50 mL of oleogel and oleofoam, respectively. All measurements were performed in triplicate.

### 4.11. Statistical Analysis

Data were analyzed using Student’s *t*-tests (Microsoft^®^ Excel^®^, Microsoft 365 (version 2205 Build 16.0.15225.20028), Redmond, Washington). The significance level was set at *p* < 0.05. The analysis of overrun study is reported in the Supplementary Materials.

## 5. Patents

G.A., E.K., E.G.A. and D.G.F. are inventors on a patent application related to this work filed by the Aristotle University of Thessaloniki: PCT Ιnternational Application No. PCT/GR2020/000024 filed on 15 May 2020 and published under No. WO2020/229851 on 19 November 2020; European Patent Application No. EP20742414.4 published under No. ΕΡ 3968954 on 23 March 2022.

## Data Availability

Not applicable.

## Supplementary Materials

The following supporting information can be downloaded at: https://www.mdpi.com/article/10.3390/gels8110705/s1, Table S1: Crystallization temperature Tc and total crystallization enthalpy ΔHc for non-isothermal conditions; Table S2: Peak melting temperatures and Tm and melting enthalpies Δ*Hm* of the different formulations after 0 h and 24 h storage time at 4 °C; Figure S1: Process cooling profile and process cooling rate (First Derivative); Figure S2: Raw materials heating curves as obtained by Differential Scanning Calorimetry for heating rate of 10 °C/min; Figure S3: Raw materials cooling curves as obtained by Differential Scanning Calorimetry for cooling rate of 10 °C/min; Figure S4: Relative crystallinity (quantified by DSC data) plotted as a function of temperature: (A) 20 GMS, (C) 20 GMS/1 GMO, (E) 20 GMS/2.5 GMO, and (G) 20 GMS/5 GMO; and as a function of time: (B) 20 GMS, (D) 20 GMS/1 GMO, (F) 20 GMS/2.5 GMO, and (H) 20 GMS/5 GMO, for non-isothermal crystallization at cooling rates from 1 to 20 °C/min; Figure S5: Half time crystallization (*t*_1/2_) of the different formulations vs cooling rate; Figure S6: Complex viscosity η* plotted as a function of temperature: (A) 20 GMS, (C) 20 GMS/1 GMO, (E) 20 GMS/2.5 GMO, and (G) 20 GMS/5 GMO. Relative crystallinity (quantified by rheology data) plotted as a function of time: (B) 20 GMS, (D) 20 GMS/1 GMO, (F) 20 GMS/2.5 GMO, and (H) 20 GMS/5 GMO, for non-isothermal crystallization at cooling rates from 1 to 20 °C/min; Figure S7: Polarized Microscopy images of the different samples, obtained directly after cooling at different cooling rates. (A) Scale bar at 20 μm and (B) scale bar at 100 μm. Linear enhancement of the same level was applied to all micrographs; Figure S8: OBC values of the different formulations, for two centrifugation times; Figure S9: MCT oil oleofoams composed of (A) 20% GMS, (B) 20% GMS/1% GMO, (C) 20% GMS/2.5% GMO, and (D) 20% GMS/5% GMO; Figure S10: Overrun values (%) after 5 min of whipping; Figure S11: Polarized Microscopy micrographs of the prepared foams directly after the whipping process. (Scale bar at 20 μm and 100 μm). Linear enhancement of the same level was applied to all micrographs; Figure S12: DSC data as they were obtained by the DSC instrument. These data were used for the analysis described by Figure 1.
