# Supplementary material for: Effect of Glyceryl Monoolein Addition on the Foaming Properties and Stability of Whipped Oleogels
Source: Gels. 2022 Oct 31;8(11):705. doi: 10.3390/gels8110705 (PMC9689941; doi:10.3390/gels8110705)
Supplement: Supplementary file 1 [file gels-08-00705-s001.zip › gels-1982207-supplementary.pdf]

gels

Supplementary Materials

Effect of Glyceryl Monoolein Addition on the Foaming Properties and Stability of Whipped Oleogels

Eleftherios G. Andriotis [1,*], Paraskevi-Kyriaki Monou [1], George Komis [2], Nikolaos Bouropoulos [3,4], Christos Ritzoulis [5], Georgios Delis [6], Evangelos Kiosis [7], Georgios Arsenos [8] and Dimitrios G. Fatouros [1]

Table S1. Crystallization temperature *Tc* and total crystallization enthalpy *ΔHc* for non-isothermal conditions.

Cooling Rate	20 GMS			20 GMS/1 GMO			20 GMS/2.5 GMO			20 GMS/5 GMO		
(°C/min)	T_{c1} (°C)	T_{c2} (°C)	ΔHc_{total} (J/g)	T_{c1} (°C)	T_{c2} (°C)	ΔHc_{total} (J/g)	T_{c1} (°C)	T_{c2} (°C)	ΔHc_{total} (J/g)	T_{c1} (°C)	T_{c2} (°C)	ΔHc_{total} (J/g)
1	40.2	42.5	0.5	36.5	42.6	0.3	35.7	42.5	0.4	39.2	40.3	0.5
5	38.8	41.1	2.3	35.2	41.3	1.9	34.6	41.8	2.3	38.8	41.1	2.4
10	37.8	40.2	5.2	34.4	40.4	3.9	33.9	40.7	4.8	36.5	39.0	5.3
20	37.3	40.0	4.5	32.9	38.5	8.1	33.0	39.4	7.7	35.3	38.2	7.3

Table S2. Peak melting temperatures Tm and melting enthalpies *ΔHm* of the different formulations after 0 h and 24 h of storage time at 4 °C.

	t=0h at 4 °C		t=24h at 4 °C	
	T_m (°C)	ΔH_m (J/g)	T_m (°C)	ΔH_m (J/g)
20 GMS	47.5	-23.04	54.2	-22.53
20 GMS/1 GMO	44.6	-22.27	42.7	-13.69
20 GMS/2.5 GMO	44.1	-37.07	42.8	-24.7
20 GMS/5 GMO	45.7	-25.21	54.5	-23.87

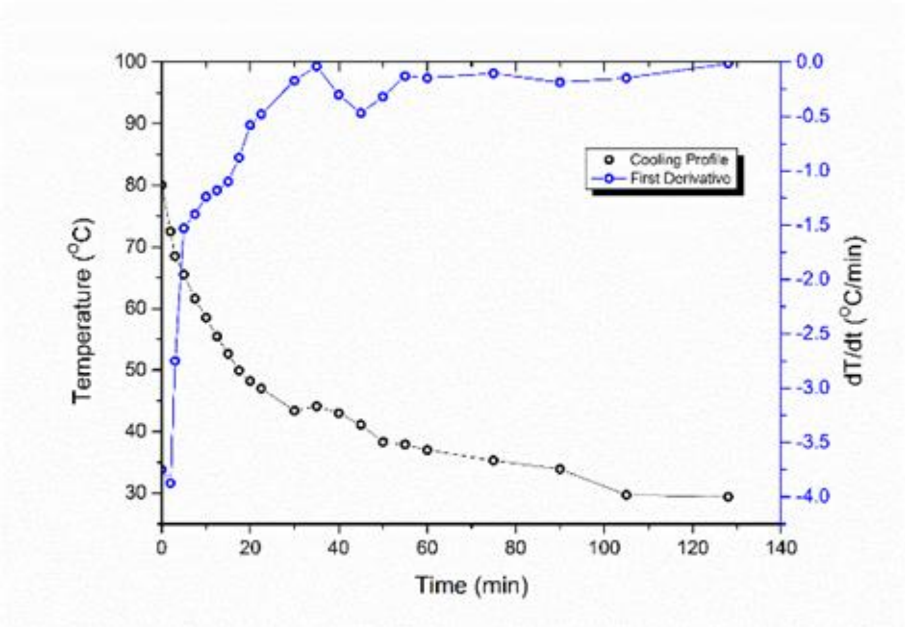

Figure S1. Process cooling profile and process cooling rate (first derivative).

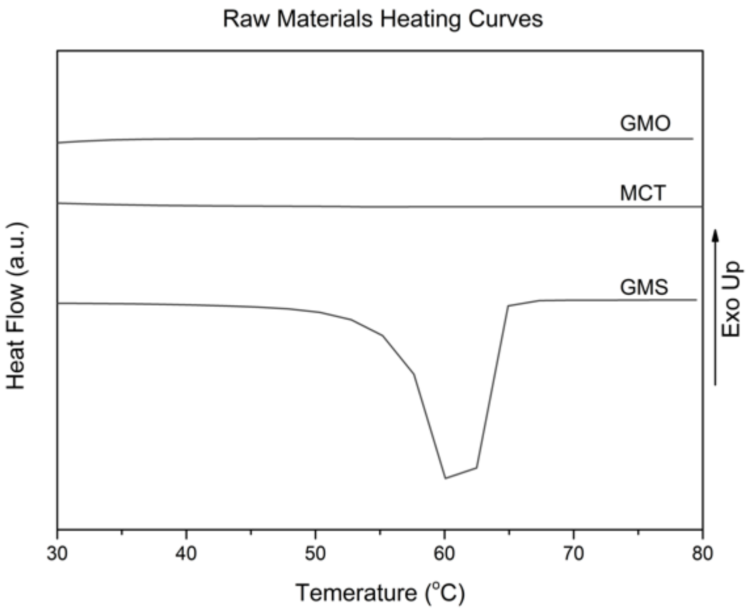

Figure S2. Raw material heating curves as obtained by Differential Scanning Calorimetry for a heating rate of 10 °C/min.

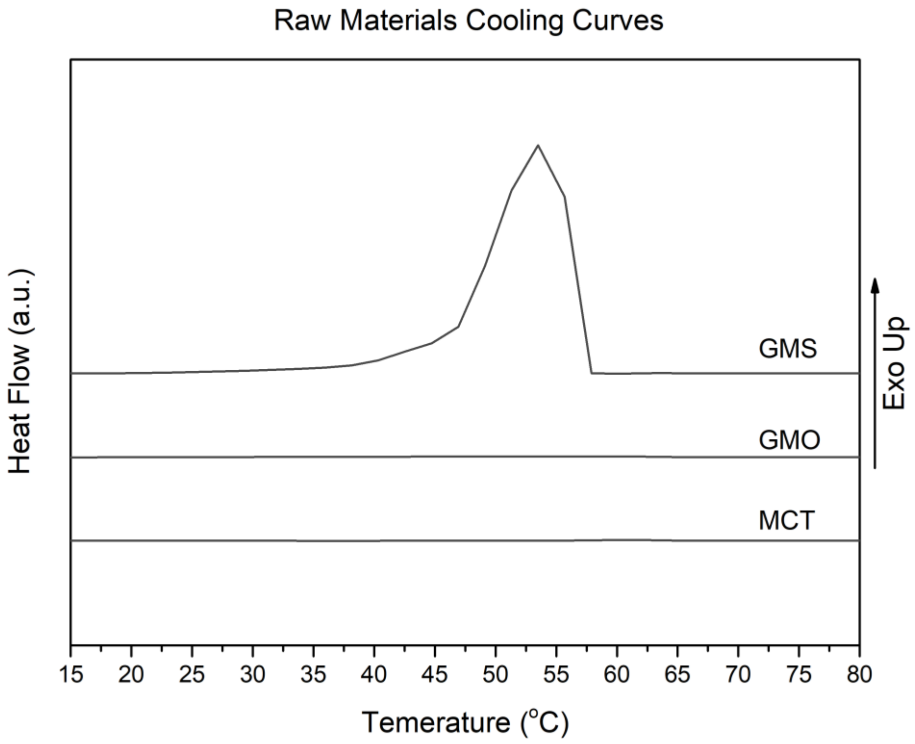

Figure S3. Raw material cooling curves as obtained by Differential Scanning Calorimetry for a cooling rate of 10 °C/min.

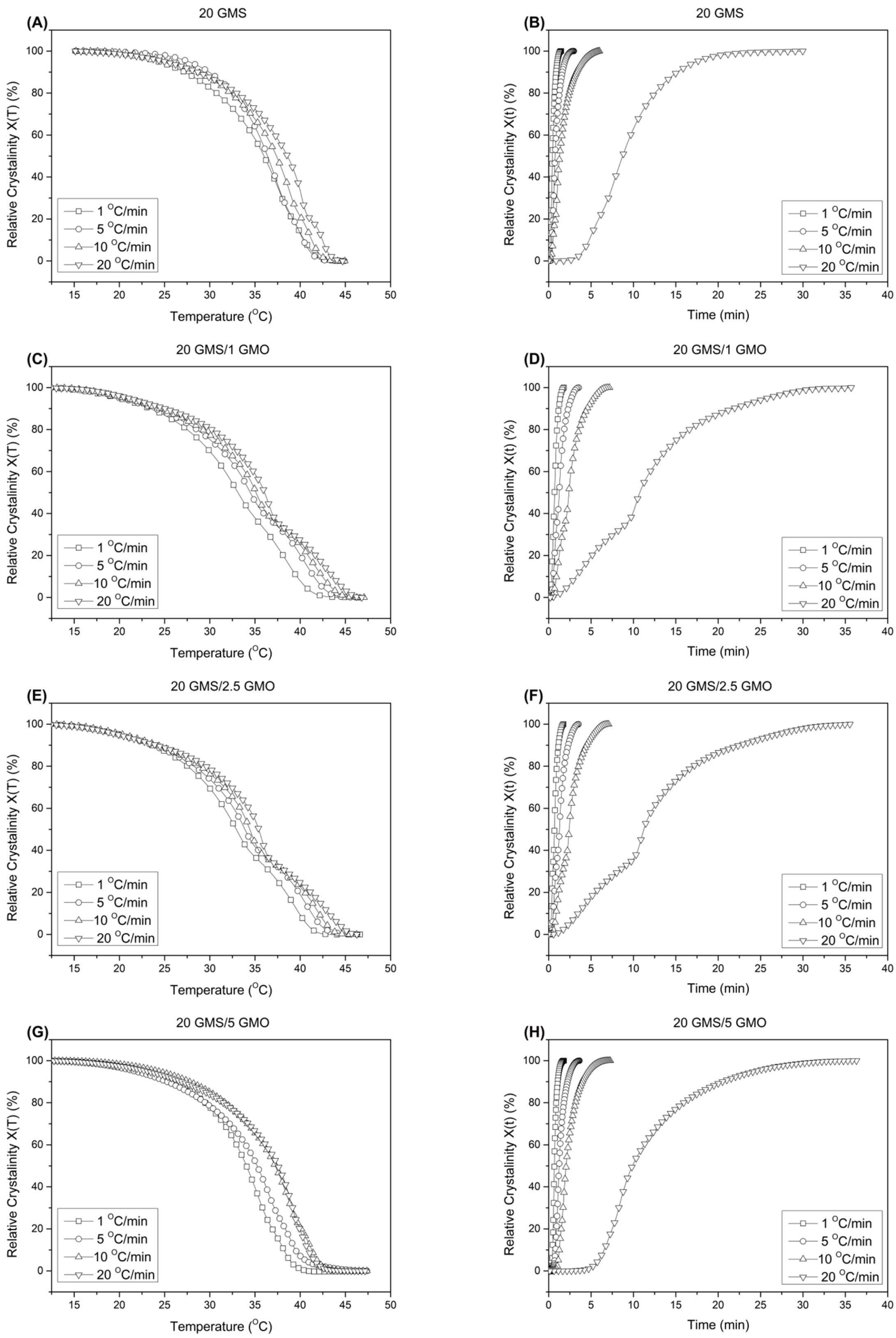

Figure S4. Relative crystallinity (quantified by DSC data) plotted as a function of temperature ((A) 20 GMS, (C) 20 GMS/1 GMO, (E) 20 GMS/2.5 GMO, and (G) 20 GMS/5 GMO) and as a function of time ((B) 20 GMS, (D) 20 GMS/1 GMO, (F) 20 GMS/2.5 GMO, and (H) 20 GMS/5 GMO) for non-isothermal crystallization at cooling rates from 1 to 20 °C/min.

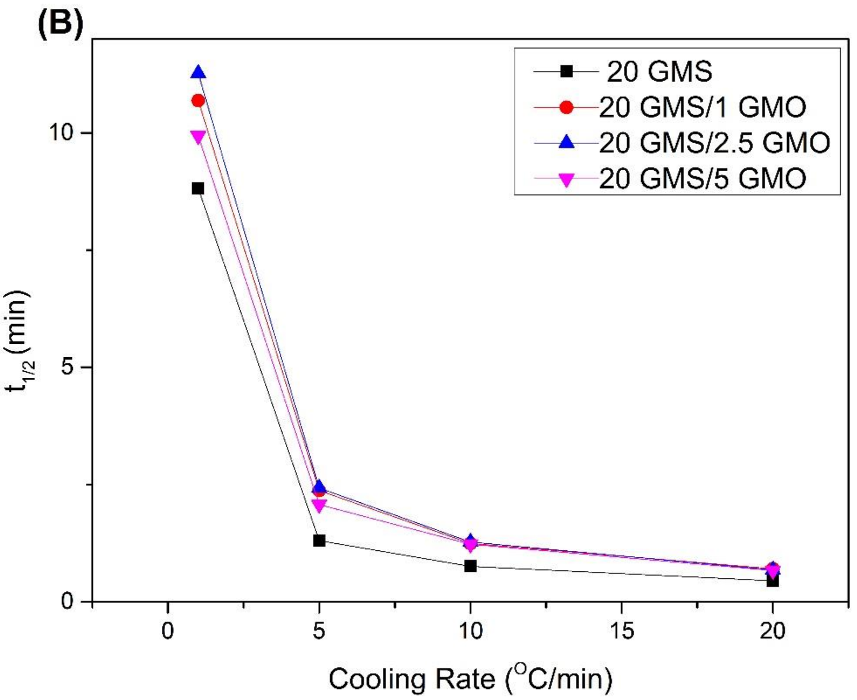

Figure S5. Half-time crystallization ($t_{1/2}$) of the different formulations vs. cooling rate.

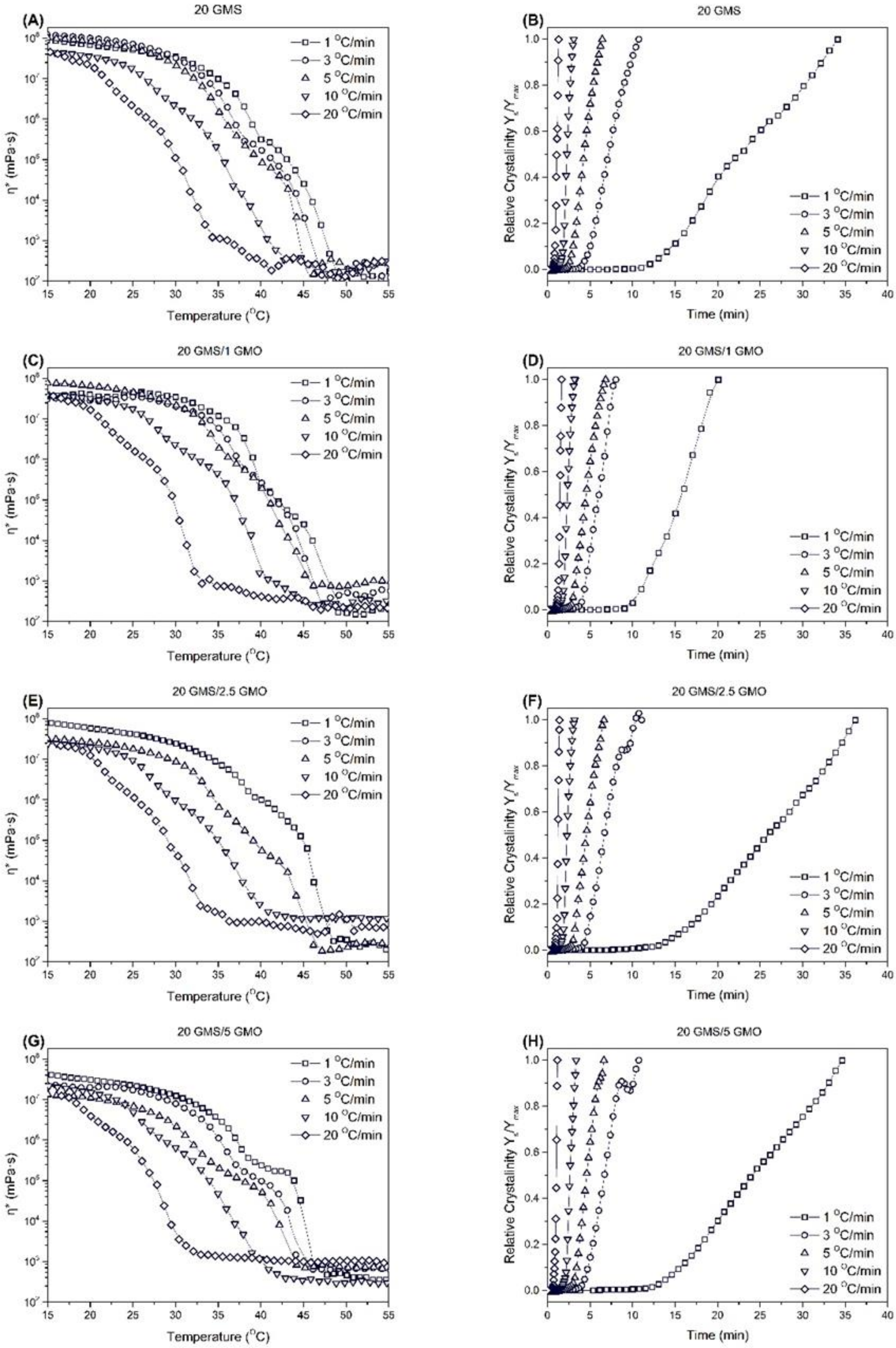

Figure S6. Complex viscosity η* plotted as a function of temperature: (A) 20 GMS, (C) 20 GMS/1 GMO, (E) 20 GMS/2.5 GMO, and (G) 20 GMS/5 GMO. Relative crystallinity (quantified by rheology data) plotted as a function of time for non-isothermal crystallization at cooling rates from 1 to 20 °C/min: (B) 20 GMS, (D) 20 GMS/1 GMO, (F) 20 GMS/2.5 GMO, and (H) 20 GMS/5 GMO.

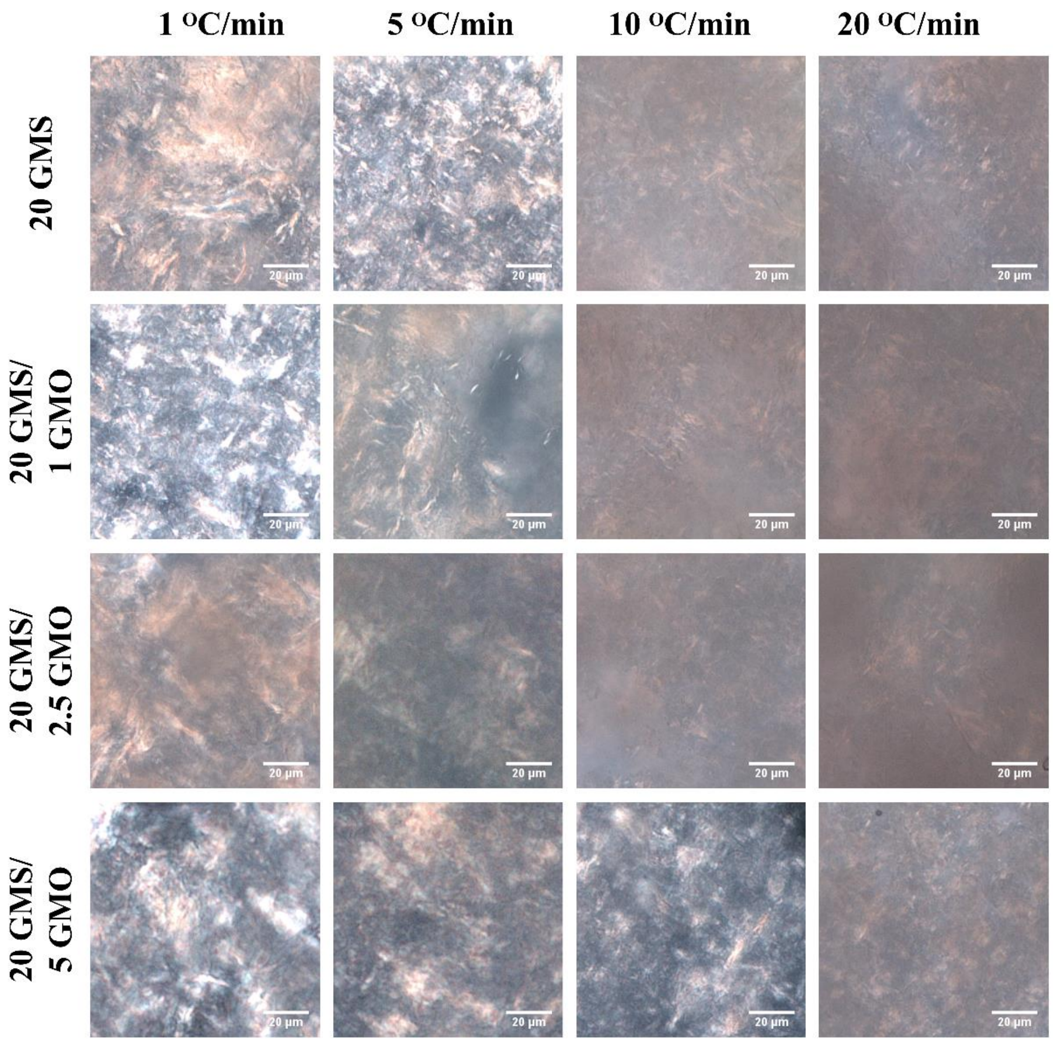
1 °C/min
5 °C/min
10 °C/min
20 °C/min
20 GMS
20 GMS/ 1 GMO
20 GMS/ 2.5 GMO
20 GMS/ 5 GMO
20 μm

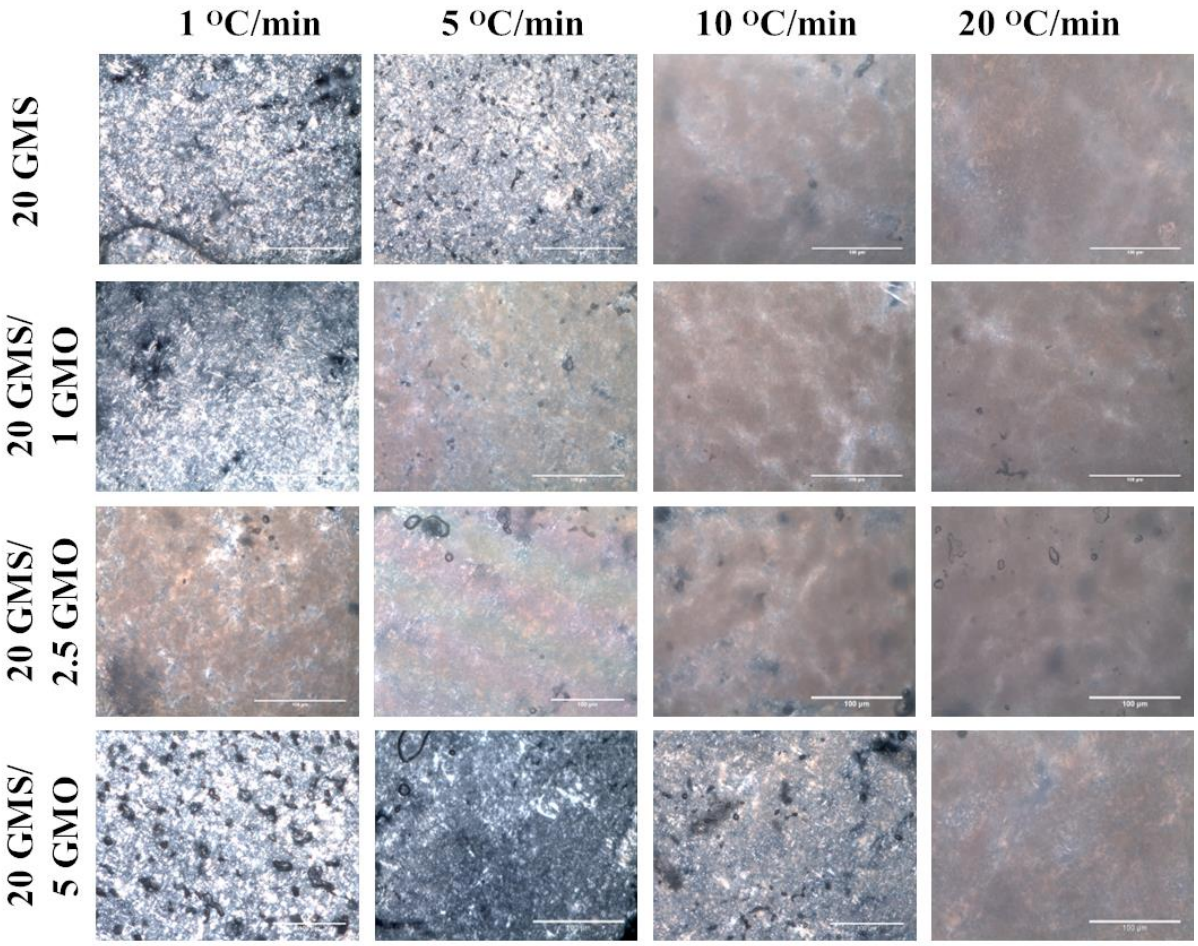

Figure S7. Polarized microscopy images of the different samples obtained directly after cooling at different cooling rates. (A): scale bar of 20μm. (B): scale bar of 100μm. The same level of linear enhancement was applied to all micrographs.

Oil-Binding Capacity (OBC). Prior to any evaluation of the foaming process, the whipped and un-whipped samples were evaluated for their Oil-Binding Capacity. The OBC test was performed in two steps including 15 min and 30 min centrifugation cycles. These two cycles represent short- and intermediate-term stability simulations. OBC is used to determine the effect of the foaming process on the physical stability of the system. It is indicative of the ability of the crystalline matrix to entrap the oil phase efficiently [1]. The effect of the foaming process on OBC is shown in Figure S8A-B for both the first and second centrifugation cycles. According to Figure S9, the foaming process induces a phase separation which is intensified by 30 min of centrifugation. This observation is expected as the produced un-whipped samples (oleogels) are subjected to a low shear process that partially influences the mechanical cohesion of the gel, causing oil drainage. On the other hand, the addition of GMO in the formulations leads to a more stable foam in terms of OBC, with 1% w/w addition being the optimum formulation.

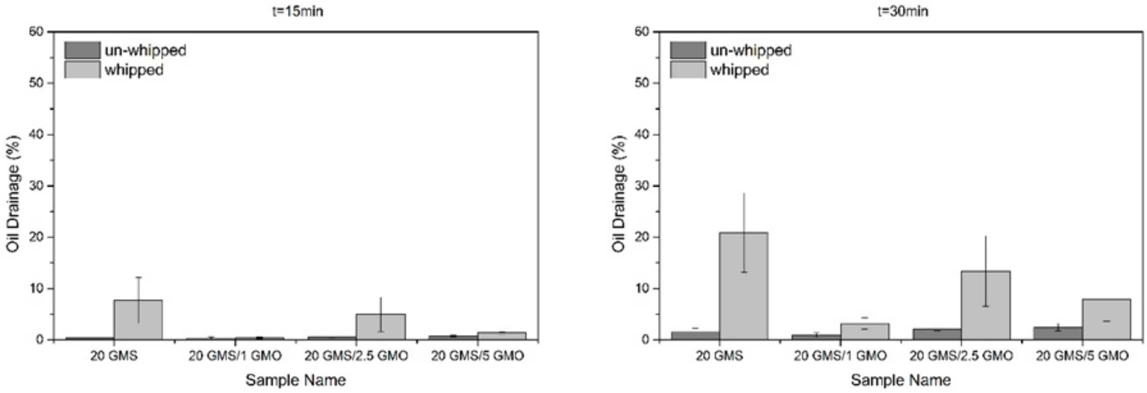

Figure S8. OBC values of the different formulations for the two centrifugation durations.

Foamability Evaluation. The foaming process, as has previously been described, resulted in the formation of homogeneous viscous foams that have a dense white opaque appearance (Figure S9), which is in accordance with similar systems that have been reported in the literature [2]. The produced foams were evaluated in terms of the amount of air entrapped within the bulk of the material after 5 min of whipping. Figure S10 reveals that the incorporation of GMO in the formulations had no apparent effect on the measured overrun for the selected whipping duration. The relatively low overrun values measured are in close agreement with the literature for similar whipping times [2].

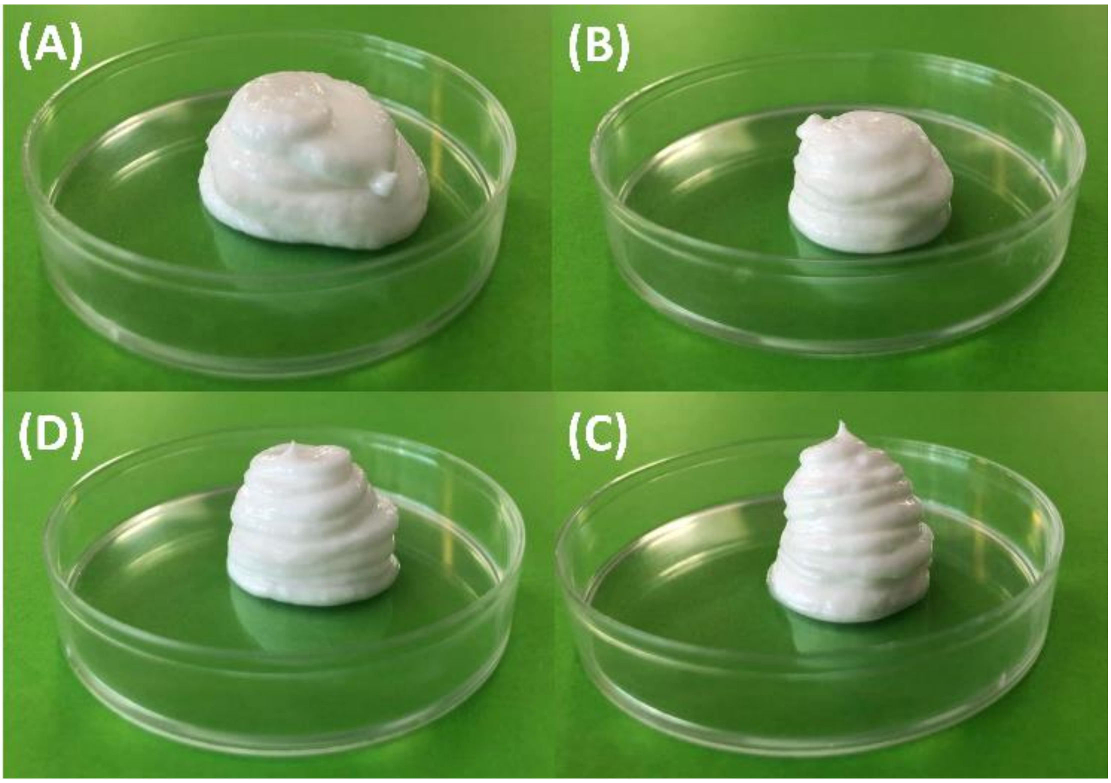

Figure S9. MCT oil oleofoams composed of (A) 20% GMS, (B) 20% GMS/ 1% GMO, (C) 20% GMS/ 2.5% GMO, and (D) 20% GMS/ 5% GMO.

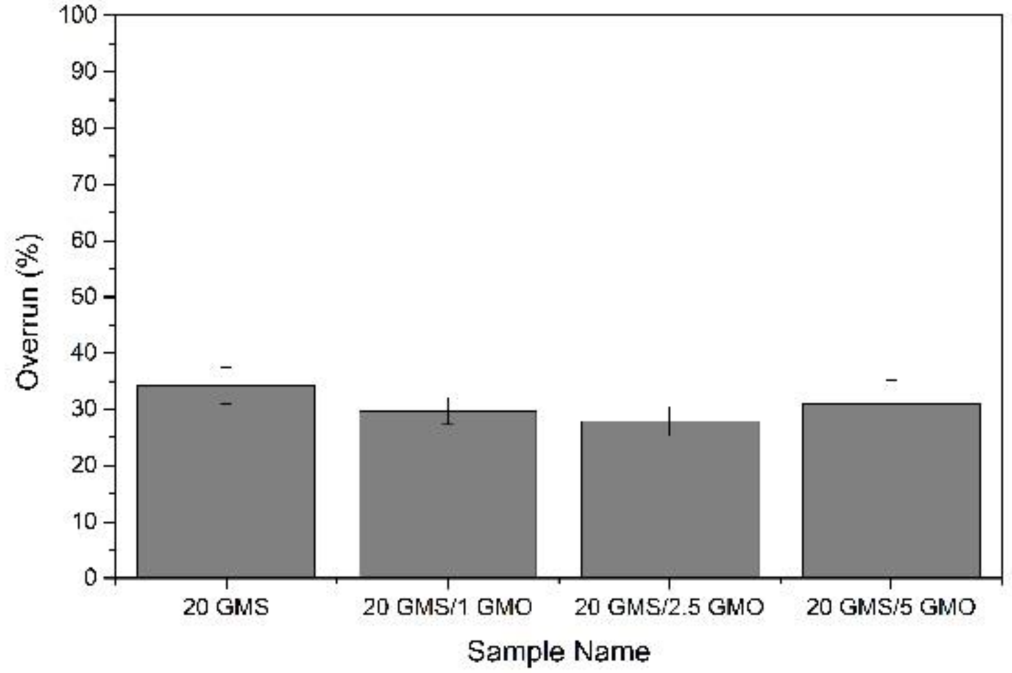

Figure S10. Overrun values (%) after 5 min of whipping.

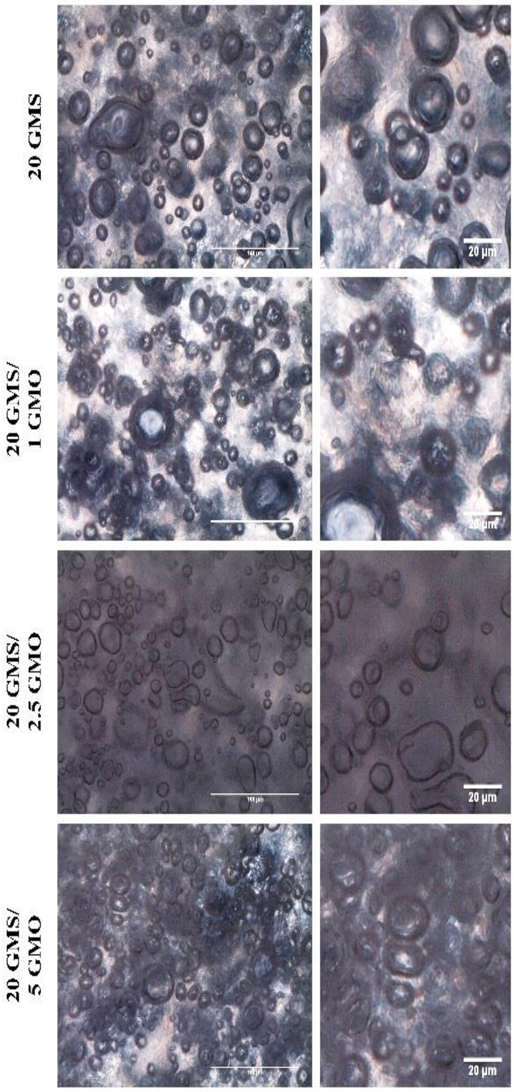

Figure S11. Polarized microscopy micrographs of the prepared foams directly after the whipping process (scale bars of 20μm and 100μm). The same level of linear enhancement was applied to all micrographs.

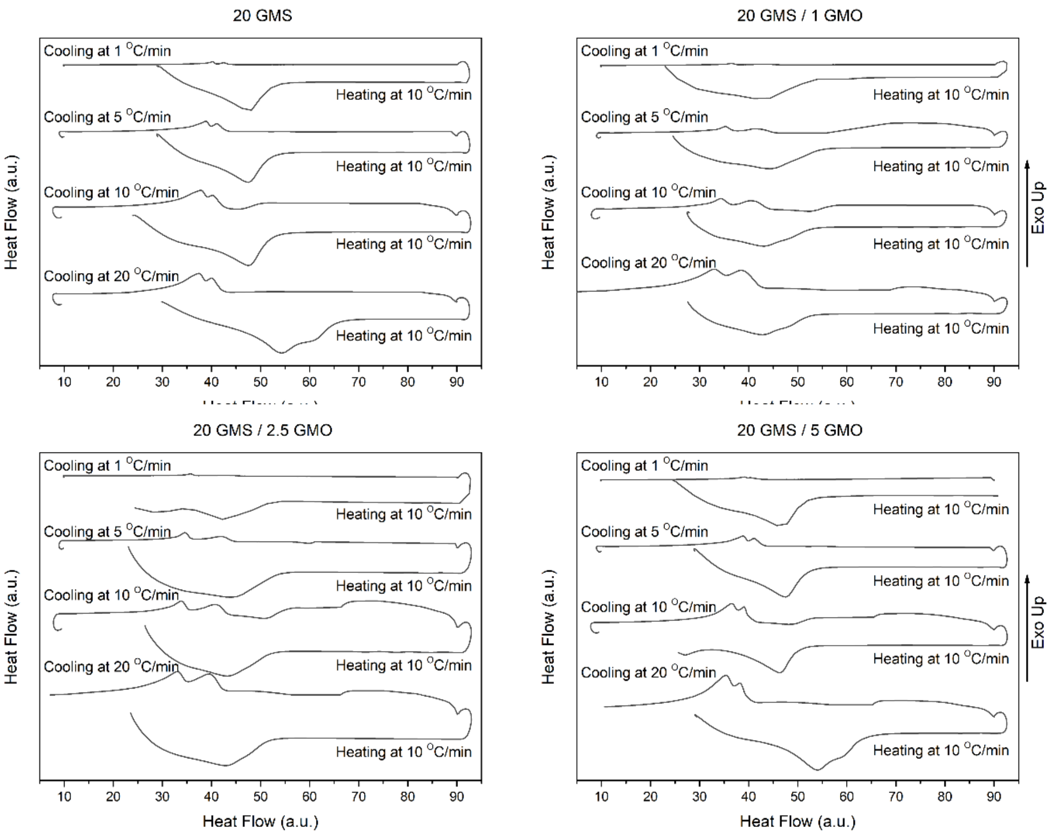

Figure S12. DSC data as they were obtained by the DSC instrument. These data were used for the analysis described in Figure 1.
